# Unusual Sciatic Nerve Entrapment by the Inferior Gluteal Artery

**DOI:** 10.3390/diagnostics16111668

**Published:** 2026-05-28

**Authors:** Wei-Ting Wu, Yu-Chun Hsu, Ke-Vin Chang, Levent Özçakar

**Affiliations:** 1 Department of Physical Medicine and Rehabilitation, Community and Geriatric Research Center, National Taiwan University Hospital, Bei-Hu Branch, Taipei 108206, Taiwan; wwtaustin@yahoo.com.tw (W.-T.W.); viph062@gmail.com (Y.-C.H.); 2 Department of Physical Medicine and Rehabilitation, National Taiwan University College of Medicine, Taipei 100233, Taiwan; 3 Center for Regional Anesthesia and Pain Medicine, Wang-Fang Hospital, Taipei Medical University, Taipei 110301, Taiwan; 4 Department of Physical and Rehabilitation Medicine, Hacettepe University Medical School, Ankara 06100, Turkey; lozcakar@yahoo.com

**Keywords:** ultrasonography, sciatica, compression, gluteus, hydrodissection

## Abstract

Sciatic neuropathy is most commonly attributed to spinal or muscular causes, whereas vascular-related compression remains underrecognized. We report a case of sciatic nerve entrapment caused by an anomalous inferior gluteal artery in the deep gluteal region, who presented with persistent right gluteal and posterior thigh pain for more than two years, refractory to multiple conservative treatments. Physical examination demonstrated marked allodynia and a well-defined Tinel-like sign, with radiating symptoms extending to the lower limb, suggesting a peripheral etiology. High-resolution ultrasonography identified an aberrant inferior gluteal artery, which crossed over and compressed the sciatic nerve, forming an accompanying artery of the sciatic nerve. Doppler imaging confirmed the vascular nature of the structure, while long-axis views demonstrated focal nerve compression with segmental swelling. Magnetic resonance imaging further corroborated the diagnosis. Ultrasound-guided hydrodissection using 5% dextrose and lidocaine was performed, resulting in significant symptom relief. Pain scores improved from 7 to 3 after treatment, with resolution of symptoms at two-month follow-up. This case highlights a rare neurovascular cause of sciatic nerve entrapment and underscores the importance of ultrasonography in identifying anatomical variations. Recognition of vascular contributions to deep gluteal syndrome may improve diagnostic accuracy and guide targeted interventions.

**Figure 1:**
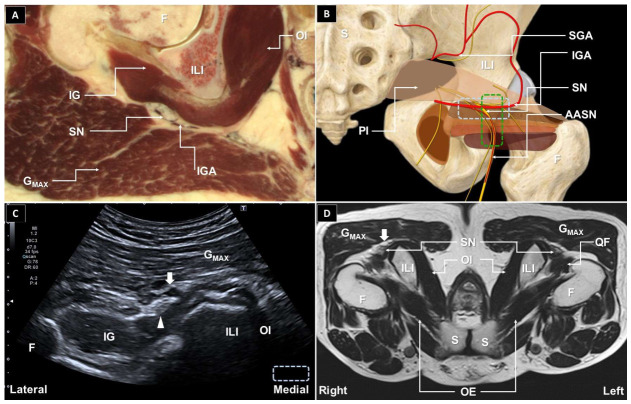
The cross-sectional cadaveric image from the Visible Human Project^®^ (used with permission from Touch of Life Technologies Inc.) (**A**) and illustration (**B**) of the anatomical relationship between the inferior gluteal artery and sciatic nerve in the gluteal region. Short-axis ultrasonographic image (**C**) and the corresponding axial T2-weighted magnetic resonance image (**D**) demonstrate engorgement of the inferior gluteal artery on the right side (white arrow), with associated sciatic nerve entrapment, compared with the contralateral side. SGA, superior gluteal artery; IGA, inferior gluteal artery; SN and white arrowhead, sciatic nerve; AASN, accompanying artery of the sciatic nerve; PI, piriformis muscle; GMAX, gluteus maximus; IG, inferior gemellus; OI, obturator internus; QF, quadratus femoris; ILI, ilium; F, femur; OE, obturator externus; S, sacrum; colored dashed box, transducer’s positions.

**Figure 2:**
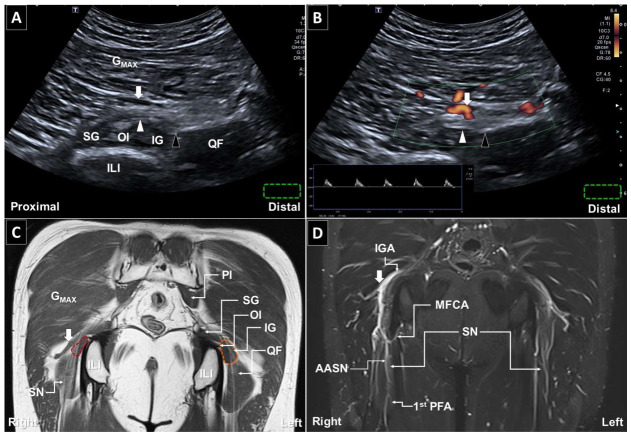
Long−axis ultrasonographic B−mode (**A**) and Doppler−mode (**B**) images demonstrate sciatic nerve entrapment by the inferior gluteal artery (white arrow). The site of entrapment (white arrowhead) and focal swelling of the sciatic nerve (black arrowhead) are seen. Corresponding coronal T1−weighted (**C**) and contrast-enhanced T1−weighted (**D**) magnetic resonance images demonstrate engorgement of the inferior gluteal artery on the right side (white arrow) compared with the contralateral side (orange dashed line), accompanied by atrophy of the inferior gemellus muscle (red dashed line). IGA, inferior gluteal artery; SN, sciatic nerve; AASN, accompanying artery of the sciatic nerve; PI, piriformis muscle; GMAX, gluteus maximus; SG, superior gemellus; IG, inferior gemellus; OI, obturator internus; QF, quadratus femoris; ILI, ilium; MFCA, medial circumflex femoral artery; 1st PFA, first perforating artery. The ultrasonographic images were obtained from a 50−year-old man who presented with persistent tightness and pain in the right gluteal region and posterior thigh for more than two years. According to his description, the symptoms had been noticeable since adolescence; however, the pain progressively worsened over the past two years with prolonged sitting at work and weight gain. Following the initiation of gym-based gluteal strengthening and bicycle training, the symptoms became more severe. The patient had no relevant comorbidities, including thyroid disease or diabetes mellitus. Previous conservative treatments, including lumbar traction and ultrasound-guided injections targeting the lumbar facet joints, piriformis muscle, and caudal epidural space, provided only limited symptomatic relief.

**Figure 3:**
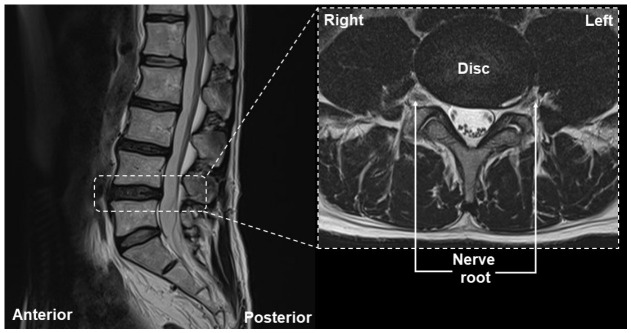
The sagittal T2-weighted magnetic resonance imaging (MRI) of the lumbar spine and axial image at the L4–L5 level demonstrate mild disc protrusion without significant nerve root or spinal canal compression. MRI of the lumbar spine revealed a small L4–L5 intervertebral disc herniation without significant nerve root compression or annular disruption on axial views. Furthermore, axial MRI images from the L3–S1 levels demonstrated no significant nerve root lesion or compression. On physical examination, the straight leg raise test was negative, with no radiating pain from the posterior thigh to the lower leg and no sensory deficits in the lower extremities. Muscle strength was preserved in both lower limbs, although the patient reported a sensation of tightness during hip internal rotation, accompanied by reduced standing and walking endurance. No remarkable gait abnormality or foot drop was observed in this patient. Deep tendon reflexes were within normal limits, with no signs suggestive of upper motor neuron involvement. Palpation over the symptomatic area elicited marked buttock pain, along with a well-defined Tinel-like sign, with radiating symptoms extending from the ischiofemoral space to the lower limb. Nerve conduction studies demonstrated no significant abnormalities in sensory conduction velocity or latency. A mild reduction in CMAP amplitudes was observed in both the tibial and common peroneal nerves. The F-wave latency was mildly prolonged compared with the contralateral side but remained within normal limits. Electromyography revealed no significant polyphasic changes in the gluteus maximus, gluteus medius, or erector spinae muscles; however, increased polyphasic activity was noted in the biceps femoris and medial gastrocnemius on the affected side. No asymmetry in gluteal muscle bulk was identified between the two sides. Musculoskeletal ultrasound examination was conducted using an Aplio i700 ultrasound system equipped with a curvilinear transducer (10C3, 3.6–9.2 MHz; Canon Medical Systems Corporation, Otawara, Japan). The transducer was initially placed over the ilium and then moved caudally until the greater sciatic notch was identified. The regional anatomy (Figure 1A) and corresponding transducer placement are illustrated in Figure 1B [1]. In this case, the inferior gluteal artery on the right side did not follow its typical course superficial to the piriformis muscle [2,3]. Instead, it coursed distally and laterally, running alongside and subsequently crossing over the sciatic nerve in the short-axis view (Figure 1C), forming the accompanying artery of the sciatic nerve [4,5]. Corresponding axial T2-weighted magnetic resonance image demonstrated engorgement of the inferior gluteal artery in the right gluteal region, resulting in compression of the sciatic nerve (Figure 1D). After rotating the transducer to align with the long axis of the sciatic nerve, ultrasonography clearly demonstrated nerve entrapment in the long-axis view (Figure 2A). Doppler imaging further confirmed the vascular nature of the compressive structure (Figure 2B). Subsequent magnetic resonance imaging, including coronal T1-weighted (Figure 2C) and contrast-enhanced T1-weighted (Figure 2D) sequences, corroborated the diagnosis of sciatic nerve entrapment by the inferior gluteal artery.

**Figure 4:**
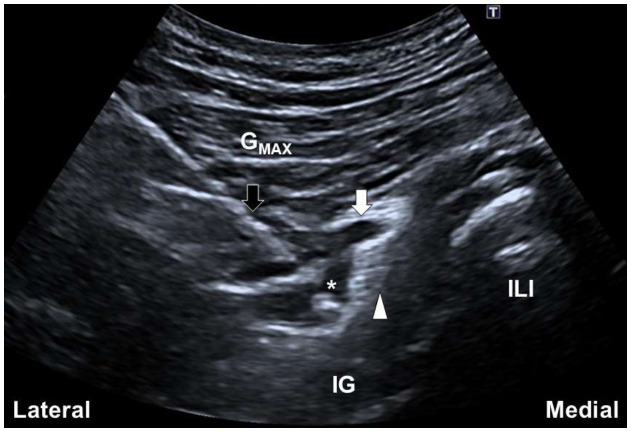
Ultrasound-guided hydrodissection of the sciatic nerve was performed using an in-plane approach, with the anechoic injectate (*) observed to distribute circumferentially around the nerve, indicating adequate separation. Black arrow, needle; white arrowhead, sciatic nerve; white arrow, engorged inferior gluteal artery. ILI, ilium; GMAX, gluteus maximus muscle; IG, inferior gemellus muscle. Based on the diagnosis of sciatic nerve irritation, ultrasound-guided hydrodissection was performed using 10 mL of 5% dextrose solution combined with 1 mL of 1% lidocaine, resulting in subsequent symptom relief. The procedure was conducted using an in-plane approach, with the needle advanced under a short-axis view of the nerve using a 23-gauge needle to release it from the surrounding musculature and the inferior gluteal artery. No procedural complications were observed. The patient’s pain improved substantially following the intervention, with the Visual Analog Scale [5] score decreasing from 7 before the procedure to 3 after treatment. At the one-month follow-up, mild recurrence of symptoms, including a sensation of tightness in the gluteal region, was reported, with the VAS score increasing to 4; therefore, a second injection was administered. At the two-month follow-up, localized tenderness and the Tinel-like sign had resolved, with the VAS score decreasing to 2. Follow-up ultrasonography demonstrated that the previously identified entrapment site had become less conspicuous. At the six-month follow-up after the initial course of treatment, no recurrence of allodynia or tenderness was noted, and both standing and walking endurance had improved compared with baseline. The sciatic nerve is the largest peripheral nerve in the human body and represents a critical structure within the posterior compartment of the lower limb. It originates from the lumbosacral plexus (L4–S3) and exits the pelvis through the greater sciatic foramen [6], most commonly inferior to the piriformis muscle [7,8]. Within the deep gluteal region, the nerve traverses a confined anatomical corridor formed by the short external rotator muscles which are arranged in a layered configuration from cranial to caudal. They include the superior gemellus, obturator internus, inferior gemellus, and quadratus femoris. This layered organization creates a narrow fibro-osseous space in which the sciatic nerve courses posterior to the muscular complex. Anatomical variations in the relationship between the sciatic nerve and piriformis muscle have been well documented. These comprise high division of the sciatic nerve, passage of one division through the piriformis muscle, or even complete penetration of the muscle belly. While deep gluteal syndrome has traditionally been attributed to muscular or fibrous entrapment, the contribution of vascular structures remains underrecognized. The inferior gluteal artery, a major branch of the internal iliac artery, exits the pelvis through the infrapiriform foramen beside the sacral plexus. The artery then courses alongside the sciatic nerve and supplies the gluteus maximus, deep gluteal muscles, and posterior thigh. After coursing laterally within the gluteal region, the inferior gluteal artery ascends to form anastomoses with the superior gluteal artery. Along this course, as observed in our case, crossing of the sciatic nerve may result in chronic neural compression and subsequent denervation. The nerve to the quadratus femoris, which also innervates the inferior gemellus muscle, may have been concomitantly involved by the lesion. However, such small neural branches are often difficult to visualize clearly on ultrasound or MRI, and their involvement could not be definitively confirmed in the present case. A particularly important vascular component is the accompanying artery of the sciatic nerve, which courses longitudinally along the medial aspect of the sciatic nerve. The arterial supply to the sciatic nerve is highly variable and may arise from the inferior gluteal artery, medial circumflex femoral artery, perforating arteries, or internal pudendal artery [4]. Multiple nutrient vessels, typically ranging from four to eight, contribute to the vascularization of the nerve and form an extensive intraneural vascular network that is essential for maintaining endoneurial homeostasis. During normal vascular development, this embryonic artery typically regresses and contributes to the formation of the inferior gluteal artery following the development of the superficial femoral artery, whereas persistence as a sciatic artery is a rare anatomical variant. Vascular structures may contribute to sciatic nerve compression through several mechanisms. Enlarged or tortuous arteries, such as a hypertrophied inferior gluteal artery or an aneurysm from persistent sciatic artery, may exert a direct mass effect on the nerve. Aberrant arterial courses, particularly those passing through or adjacent to the piriformis muscle, may create additional entrapment sites analogous to muscular compression. Furthermore, direct penetration of the sciatic nerve by arterial branches represents a rare but distinct form of neurovascular entrapment [9], in which arterial pulsation or structural distortion may result in chronic mechanical irritation of nerve fibers. In our case, repetitive friction, traction, and chronic mechanical stress related to prolonged sitting and strengthening exercises may have induced focal thickening, fibrosis, or swelling of the involved structure, such as the gluteus maximus muscle and subcutaneous tissue, ultimately forming a space-occupying lesion capable of compressing the adjacent nerve. Advanced imaging modalities, including MR neurography and angiography, may facilitate the identification of such neurovascular anomalies and improve diagnostic accuracy. Awareness of these vascular variations is therefore essential, particularly in surgical and interventional procedures involving the posterior hip, to minimize the risk of iatrogenic injury. High-resolution ultrasonography has emerged as a valuable adjunct for evaluating anatomical variations in the inferior gluteal artery and the sciatic nerve. Short-axis imaging, combined with side-to-side comparison, enhances the detection of asymmetry, while Doppler imaging facilitates differentiation of the anechoic artery from adjacent venous structures. In cases of entrapment, the affected nerve segment may appear focally narrowed, whereas the proximal and distal portions often demonstrate fusiform enlargement on long-axis views. A thorough understanding of regional anatomy, together with ultrasound-guided interventions, may aid in symptom relief and help minimize the risk of iatrogenic injury during diagnostic or therapeutic procedures [10,11,12]. The therapeutic effect of ultrasound-guided hydrodissection is primarily attributed to mechanical separation of the nerve from adjacent structures. When a nerve lies adjacent to an enlarged or pulsatile artery, chronic irritation may induce epineurial thickening and fibrotic adhesion formation. Hydrodissection can mechanically release these adhesions and reduce tethering between the nerve and surrounding vessels or soft tissues, thereby alleviating symptoms despite persistent nerve enlargement. Consequently, even after gradual absorption of the injectate, the previously entrapped or adherent segment may remain mechanically released, potentially explaining the sustained reduction in the patient’s pain. In addition, the pharmacological effects of 5% dextrose injection may also contribute to symptom relief. Elevated extracellular glucose concentrations can hyperpolarize nociceptive C-fibers and stabilize neural activation, thereby reducing noxious signal transduction [13]. The patient also followed recommendations to reduce prolonged sitting and modify both sitting posture and resistance-training techniques to avoid persistent compression over the gluteal region. However, the possibility of recurrent compression should be acknowledged. Continued clinical follow-up is therefore warranted, and appropriate postural modifications, along with the use of a suitable cushioning device, should be recommended. In cases of frequent symptom recurrence or an inadequate response to repeated hydrodissection, surgical intervention such as nerve transposition, may need to be considered.

## Data Availability

The original contributions presented in the study are included in the article, further inquiries can be directed to the corresponding author.
